# Zootechnical data analysis in a breeding animal facility: tracing the patterns of mouse production

**DOI:** 10.1186/s42826-020-00082-w

**Published:** 2021-01-04

**Authors:** Eloiza K. G. D. Ferreira, Giovanny A. C. A. Mazzarotto, Guilherme F. Silveira

**Affiliations:** 1 Instituto Carlos Chagas- Fiocruz/PR, Curitiba, PR Brazil; 2 Laboratório de Criação e Experimentação Animal- Fiocruz/PR, Curitiba, Brazil

**Keywords:** Data science, Exploratory data analysis (EDA), Python, Laboratory animals science (LAS)

## Abstract

**Background:**

With the enactment of the Brazilian Law Arouca 11,794/2008 and Decree 6.899/2009, there has been an urgent need for changes in the processes related to animal experimentation in Brazil; in particular, there is a need for improvements in enhancements of the lab animal management. To improve the management capacity of the Lab animal facility of the Carlos Chagas Institute’s Laboratory Animals Science (LAS), BioterC software was developed and implemented in 2014 for tracking mouse laboratory colonies. Five years after the implementation of this software, we sought to analyze the information in the database originated from BioterC using the Exploratory Analysis Data methodology (EDA). This article aims to identify animal breeding patterns using a data mining tool (Data Science) with Python programming language.

**Results:**

The results show that from September 2014 to June 2019, under the license IACUC number LW- 6/17, 15.106 animals were produced. The C57BL/6, BALB/c and Swiss strains were the most frequently produced strains. The distribution of births due to crosses between these strains showed a median of 6 to 10 animals, depending on the genetic homozygosis and heterozygosis of the animal. The median number of days of mating was 35 days. In the sexing period, the records reported a median of 19 days. A total of 393 requests for animals from internal and external laboratories were registered. It was noted that approximately half of the animals produced to meet the demand for orders were discarded. Of the 15,106 animals, 38% were requested for animal experimentation, 58% were discarded and 4% did not have an outcome recorded in the data.

**Conclusions:**

This volume of data provides an initial view of the information retrieval capabilities contained in BioterC, allowing for unique breeding knowledge by installing laboratory animals.

## Background

### History of the animal model

The use of animals as a living model to achieve scientific aims has been common practice since the seventeenth century. Initially, these models were used for studies of anatomy and physiology involving dissection to analyze body functions [1]. The first Brazilian laboratory animal facility that produced animals for research was inaugurated in 1904 at the Sorotherapeutic Institute of Rio de Janeiro, which today is the current Oswaldo Cruz Foundation [2]. The changes in scientific research conduct and technological advances observed in the last 116 years have led to the need for improvements in facilities due to the increasing use of animals for teaching and research.

### Arouca law

The enactment of the Arouca Law (11,794/2008), later regulated by Decree 6899/2009, prompted an investigation of current practices, leading to changes in the processes related to animal experimentation in Brazil [3]. The legislation indicated the urgency of improvements in the management of laboratory animal facilities and the standardization of maintenance and experimental conditions. To facilitate these improvements and standardizations, the use of specific software for the management of laboratory animal facilities has become an attractive option for the storage, maintenance and retrieval of data about different animal husbandry practices.

### BioterC

With the objective of improving the capacity of the management process of the Animal Breeding and Experimentation Laboratory (LACEA) of the Carlos Chagas Institute (ICC) Fiocruz PR, BioterC software (National Institute of Industrial Property - INPI - BR 512014001421–5/Ministry of Economy) was developed and implemented in 2011. During the 9 years after its implementation, BioterC not only helped in traking breeding cages and weaning litters, but also improved management, maintaining the organization and traceability of production information, include sex, date of birth, wean and cage transfer history. This software was then replaced by a new version tracking of breeding information in 2020, with improved features from the previous version, which, in addition to meeting the needs of LACEA, is also available for sale as a management resource for other laboratory animal facilities interested in the tool. BioterC is a flexible management system with different functionalities for the management of breeding colonies. LACEA’s routine is understood as the creation of SPF (Specific Pathogen-Free), with suitable and user-friendly interfaces animal models that include different strains of mice, which may be isogenic or heterogenic animals, with physical infrastructure, equipment and specialized technical staff capable of breeding manage mouse colonies and carrying out experiments with laboratory animals [4].

### Breeding according to genetic constitution

LACEA produces colonies of animals of different strains that can be classified according to their genetics, as defined through systematic reproductive practices (isogenic or heterogenic). Heterogenic strain mice are animals that have varied genetic constitution through random mating, which prevents mating animals from being close relatives. These animals are more robust and have greater fertility, with a greater number of pups per litter; the Swiss strain is an example of this group [5]. In turn, certify an isogenic lineage, it is necessary to mate at least 20 subsequent generations of a single couple, resulting in an inbreeding coefficient of 98.6% [5]. This group has lower fertility and, as a result, a lower number of offspring. Isogenic strains are of great importance because they allow experiments to be carried out with the variability of genetic origin eliminated, thus ensuring the use of fewer animals to achieve the necessary statistical power [6].

### Data management

In the present work, the information contained in the BioterC database was explored to better understand LACEA routines, with the aim of optimizing procedures. Therefore, an approach that has been widely used is Data Science, which is based on computational, mathematical, and statistical knowledge using principles of the research area that generate the information. Data Science seeks the extraction of patterns contained in the data and the application of quantitative and qualitative methods to determine the predictability of problems and results.

The use of the Data Science tool aims to assist in the identification of parameters and general patterns of mouse breeding, especially in regard to a large volume of data stored for 5 years. The purpose of this article is not only to use this tool, but also to constantly search for relevant and reproductive results that assist in animal experimentation. In addition, this study seeks to improve animal welfare through the optimization of breeding procedures, aiming to contribute to the conscious use and reduction of the zootechnical destination of animals.

In the present study, we searched for literature in articles and books on production patterns in the breeding of mice for animal experimentation, as well as their genetics.

## Results

### The use of the BioterC software brought real gains to the routine

Among the more than 700 animal facilities registered with the National Animal Production Control Council (CONCEA), as far as we know only 2 to 3 Brazilian animal facilities are employing commercial breeding data management programs. The other animal facilities use excel spreadsheets and the lack of adherence of the technical team, which is the most responsible for feeding the data, is unanimous. Brazil started to have legislation that regulated animal experimentation only from 2008. This makes it difficult to compare our data with that of other institutions. To allow this analysis, we follow below with a brief description of LACEA.

The focus of LACEA on the animal experimentation scenario in Brazil is to foster Brazilian biomedical research through the supply of laboratory animals with sanitary and genetic quality, distribution of inputs with high added biotechnological value and acting in the training of human resources in different areas of laboratory animal science. The platform is an animal breeding and experimentation laboratory capable of producing and maintaining healthy and genetically defined mice, in addition to providing rooms for conducting experiments on animals, seeking to meet strict quality requirements. LACEA is endowed with the necessary constructive aspects in order to meet the standards of biosafety and good practices for the production of laboratory animals. The area has all requirements able to classify LACEA as a barrier vivarium. An example of this is the presence of well-defined spaces in the place, such as animal breeding area, experimentation area, washing area, and support and circulation rooms (quarantine room, stock rooms, freezer room, bathrooms, secretariat, corridors, among others). LACEA also has two technical floors where the park of air-cooling machines and the central air conditioning ducts are housed. Since the beginning of its operation, LACEA has routinely served six (06) laboratories at the Carlos Chagas Institute and has sporadically served - as one-off collaborations - eight (08) external institutions.

The tracking of mating information, the occurrence of sick animals and destinations of discarded animals have been assessed daily after the introduction of BioterC. Before this information was analyzed once a month, due to the team’s lack of adherence to spreadsheets without a friendly interface and accessible by different devices such as notebooks, tablets and cell phones. This information has streamlined the checks for excessive maintenance of couples inside the rooms; couples with low reproductive index; animals in the stock without destination, also helping to control the validity and quantity of animals within each animal use license, also reflecting on the reduction of expenses with feed, nesting material, anesthetics and better control of the technical work hour. With a global view of stocks, animals are offered to external researchers. Internally, we were able to verify that the experiments are being concluded within the deadlines proposed by the licenses that authorize the use of animals. The team has reported more confidence in knowing that we have a complete database of occurrences in the colony. The veterinarian has felt a real-time direct contribution with the descriptions of sick animals, cannibalism and occurrences of fights in the colony, helping to direct the formulation of environmental enrichment programs. BioterC, with its area of ​​management of input stocks, has guaranteed predictability with the use of materials. The use of BioterC has served with support, assisted the animal facility manager in verifying the routine activities performed by each technician since the individualized login allows to verify the actions being performed by each technician inside the clean rooms.

After using BioterC, it was possible to make the communication between the team of technicians and the veterinarian clearer, which lineages and how often discarded by fights were reported. Based on this, internal campaigns were carried out at the institute to collect environmental enrichment materials, through the dissemination of digital lists about the need to improve the live conditions of the animals, where the user researchers started to contribute, voluntarily, bringing used materials for environmental enrichment. Today the animal facility of the Carlos Chagas Institute arrived in 100% boxes containing environmental enrichment, both in the areas of breeding and experimentation. After the environmental management program reformulation, there was a reduction in the occurrences of cannibalism and fights in the C57 Bl / 6 lines, as well as an increase in maternal care in the NZB line (Not shown in the work).

### The most frequently produced lines by LACEA are C57BL/6, BALB/c and Swiss

**Fig. 1:**
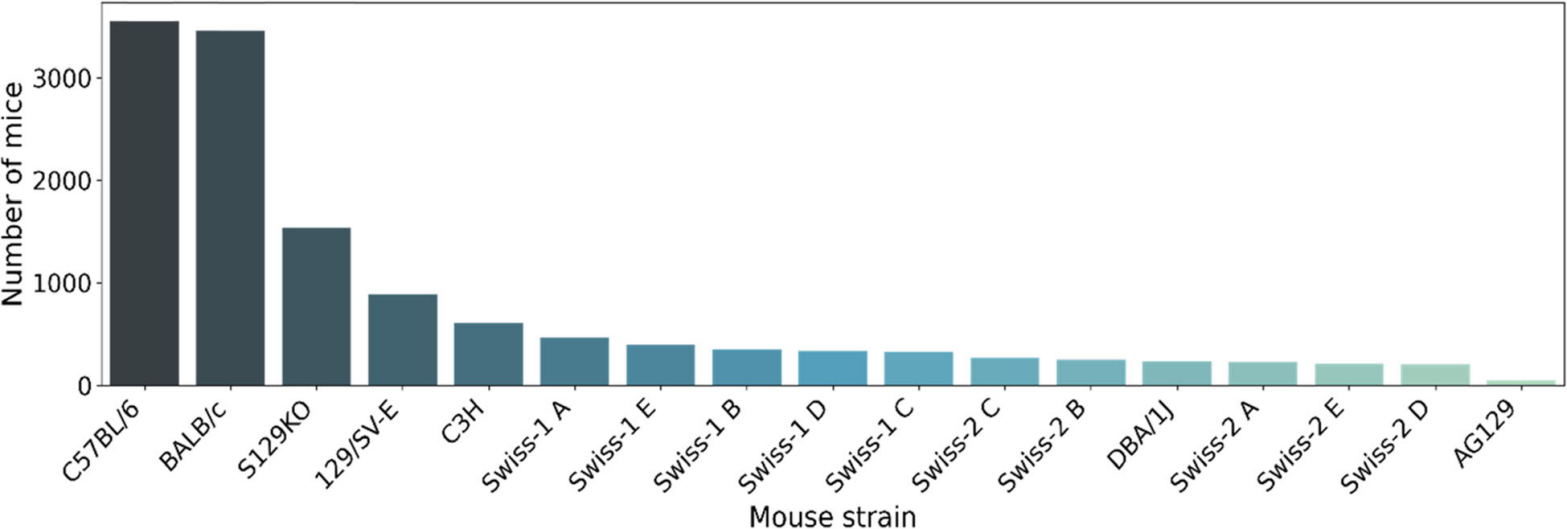
Mice produced at LACEA from Sep 2014 to Jun 2019. Total sums of mice recorded in BioterC separated by each strain produced. The C57BL/6 and BALB/c strains were the most frequently produced strains, with a difference of 101 mice produced

In 5 years, 15,106 mice were recorded in BioterC. The analysis of the strains shows that the most highly produced strains were C57BL/6 (3553 mice), BALB/c (3,452 mice) and Swiss (3025 mice) (Fig. 1).

### The isogenic group has a lower median birth rate than the heterogenic group

**Fig. 2:**
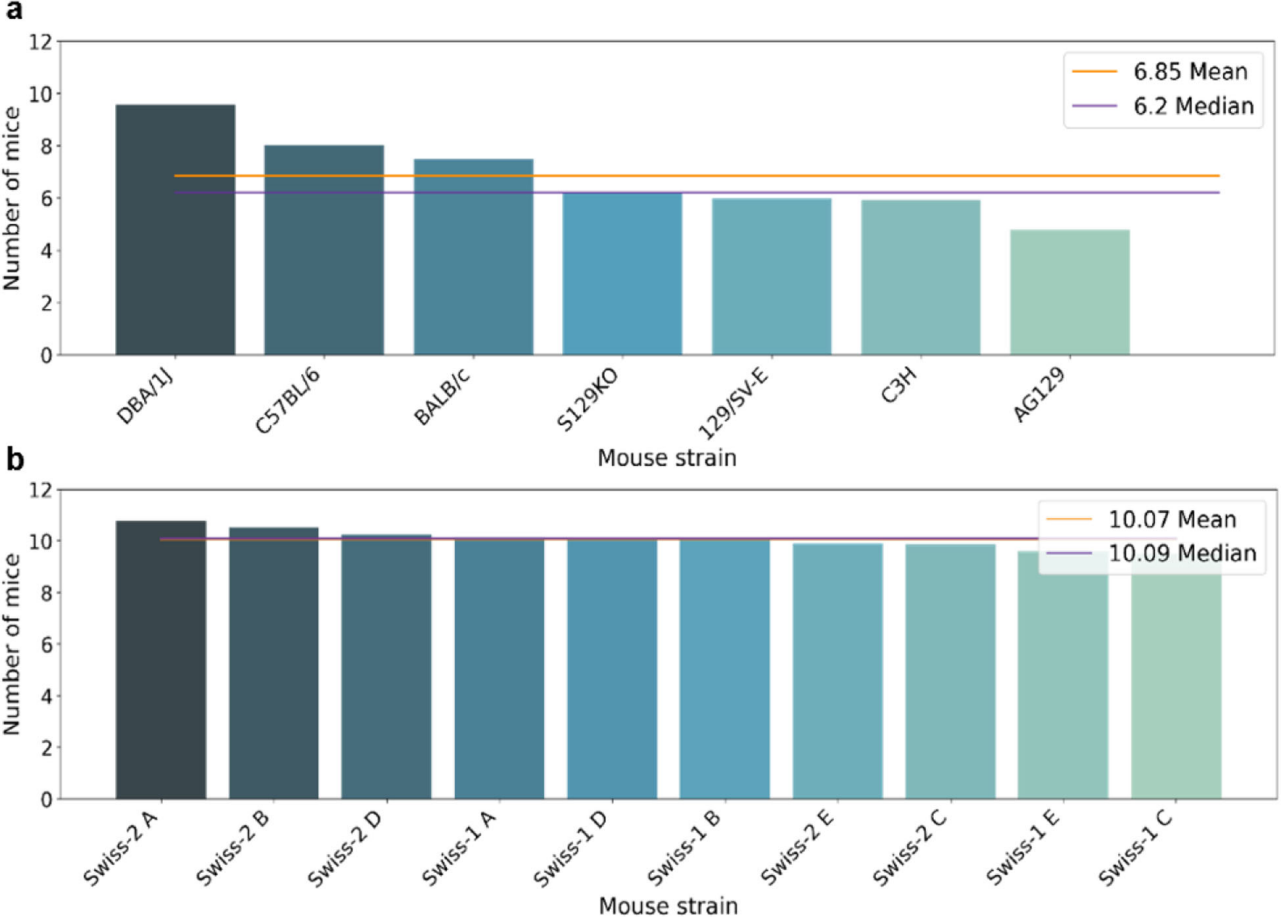
Median calculation based on birth records of the mice of different strains. **a** The group of isogenic strains has a smaller number of offspring compared to the group of heterogenic strains. **b** The Swiss (heterogenic) strain presents higher medium and median values than the Isogenic strains

When calculating the median births per group of strains according to their genetics, different values were obtained for isogenic and heterogenic mice. For isogenic strains (Fig. 2a), the data present an average of 6.85 and a median of 6.2 animals. Strains such as the DBA/1 J, C57BL/6 and BALB/c strains exhibit above average and median values, suggesting the production of more offspring pups by these strains. For the heterogenic lineage (Fig. 2b), the mean and median are very close to approximately 10.09 pups per birth.

### The median period between mating at birth is approximately 35 days

Animals of all lines from the animal facility have been placed for mating at 45 days of age, except C57/BL6 line which has been placed at 60 days of age. Today, BioterC carries information about the birth date of the animal used in the stock, the date it was placed for mating, as well as the date on which the birth of the litter is recorded. With this information, it would be possible to calculate the zootechnical indices of the interval between births, the most appropriate age of couples where the highest birth rate in each strain is observed. Nowadays, BioterC does not express these indexes directly employing graphs or reports, a situation that should be circumvented by the addition of fields in the system that make it possible to extract reports from various zootechnical indexes.

**Fig. 3:**
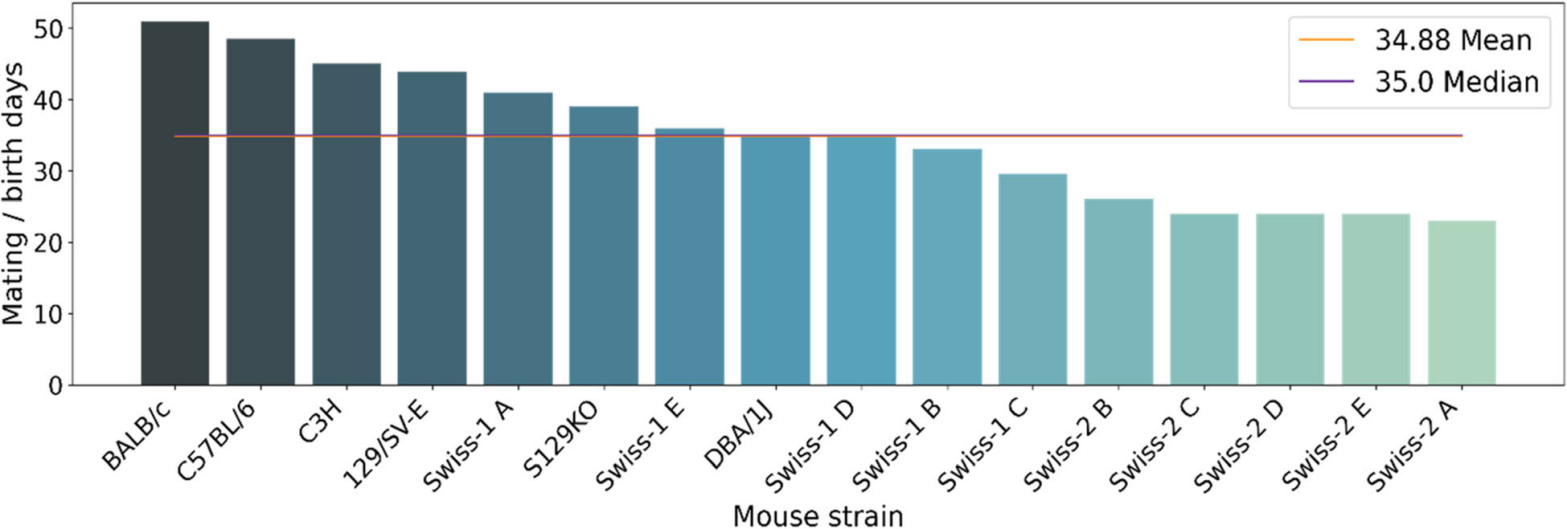
Period between the mating of mice until the birth of offspring. Most isogenic strains show higher than the average and median values between this period. The heterogenic lineages require approximately 22 to 30 days for the birth of the offspring

The period of birth is calculated based on the median between the day when mating begins until the birth of mice (Fig. 3). All isogenic strains, except for DBA/1 J, exceeded the mean (34.88) and median (35,0) of approximately 35 days from mating initiation to birth. This raises the hypothesis that it is common for isogenic groups to be maintained for a longer period of mating, as they produce a smaller number of offspring. Another hypothesis is that male mice are most often inexperienced and require longer times to mate.

### The median period between birth and sexing of mice is 19 days

**Fig. 4:**
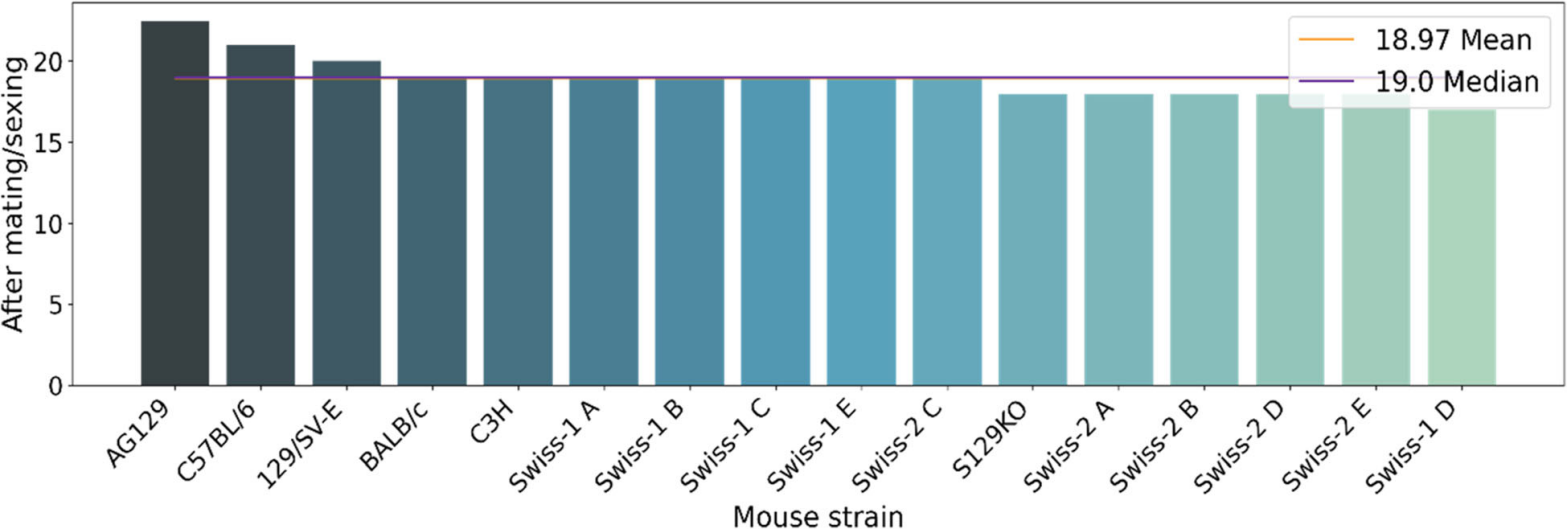
Period between the birth of the pups until the moment of sexing. Most strains are weaned within a 19-day period. Only the AG129, C57BL/6 and 129/SE-E lineages exceed this timeframe

The sexing period is calculated from birth to weaning (Fig. 4). Among all the strains, the median of this period is 19 days. When analyzing the graph, it is possible to observe that some of the strains are below or very close to the average, suggesting that these animals are being separated precociously from their mother before weaning.

### The most frequently requested strains among LACEA internal and external users are C57BL/6 and BALB/c

Seeking to better understand the flow of animal care applicants to LACEA, the data were divided between internal requests from all laboratories of the Carlos Chagas Institute and external requests from other research institutions.

**Fig. 5:**
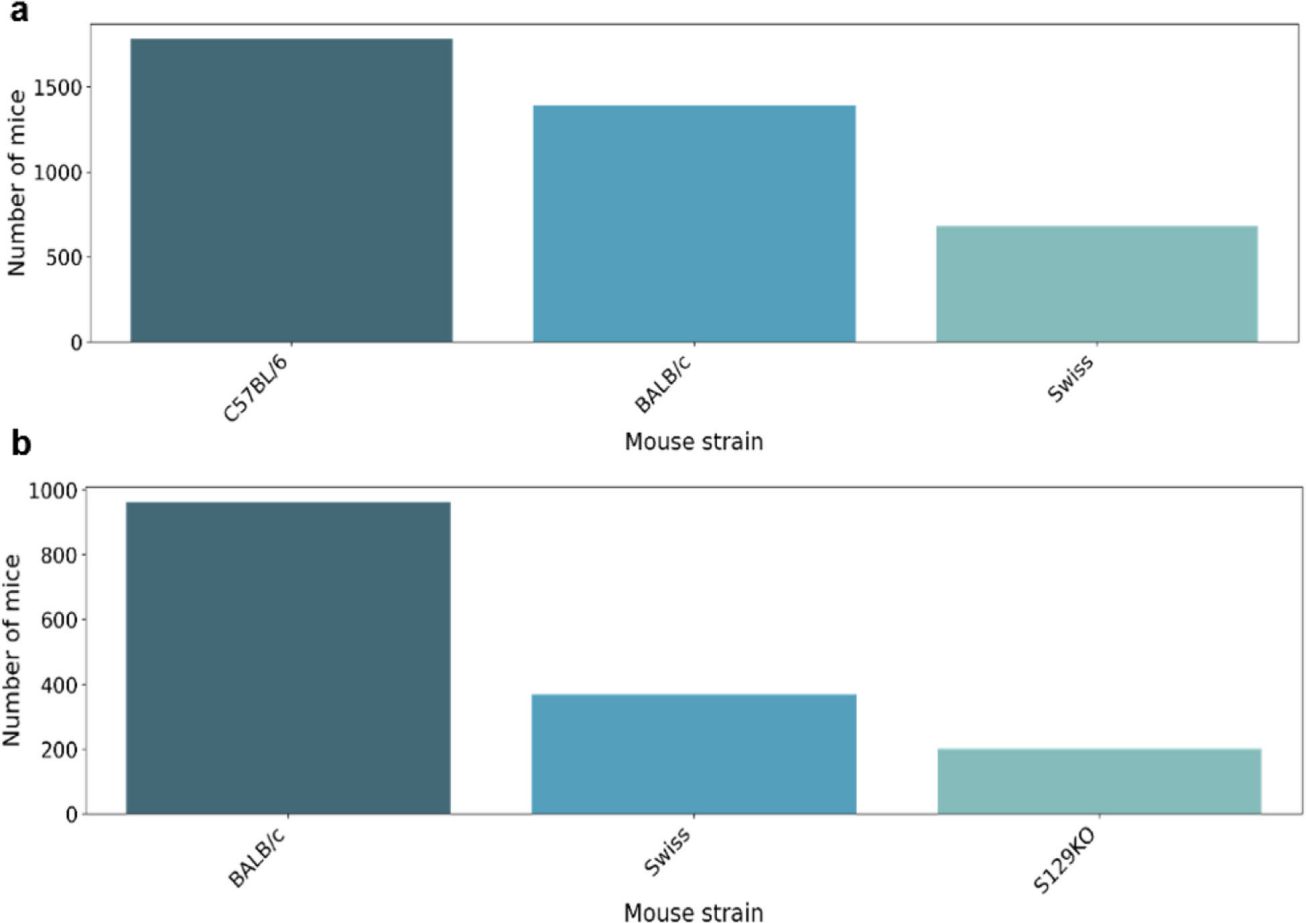
Requests for mice produced by LACEA. **a** Strains most frequently requested by the internal researchers of the Carlos Chagas Institute. BALB/c is the most frequently used lineage among different laboratories. **b** Strains frequently requested among users of external institutions, with strains C57BL/6 and BALB/c being supplied in greater numbers

The most frequently requested lineages by internal users are BALB/c (962 animals), Swiss (368 animals) and S129KO (202 animals) (Fig. 5a). The BALB/c strain is the most frequently requested lineage among most laboratories. The Swiss lineage stopped being requested among internal users in 2018. The S129KO was requested by only one group. Among the external requests, the strains that are the most frequently supplied are the C57BL/6 (1780 animals), BALB/c (1,392 animals) and Swiss (682 animals) (Fig. 5b) strains. As seen in Fig. 1, the most frequently produced lineage is C57BL/6, suggesting that this lineage is produced in LACEA mainly to be supplied to external users.

### The main reasons for discarding mice are zootechnical disposal and end of an experiment

**Fig. 6:**
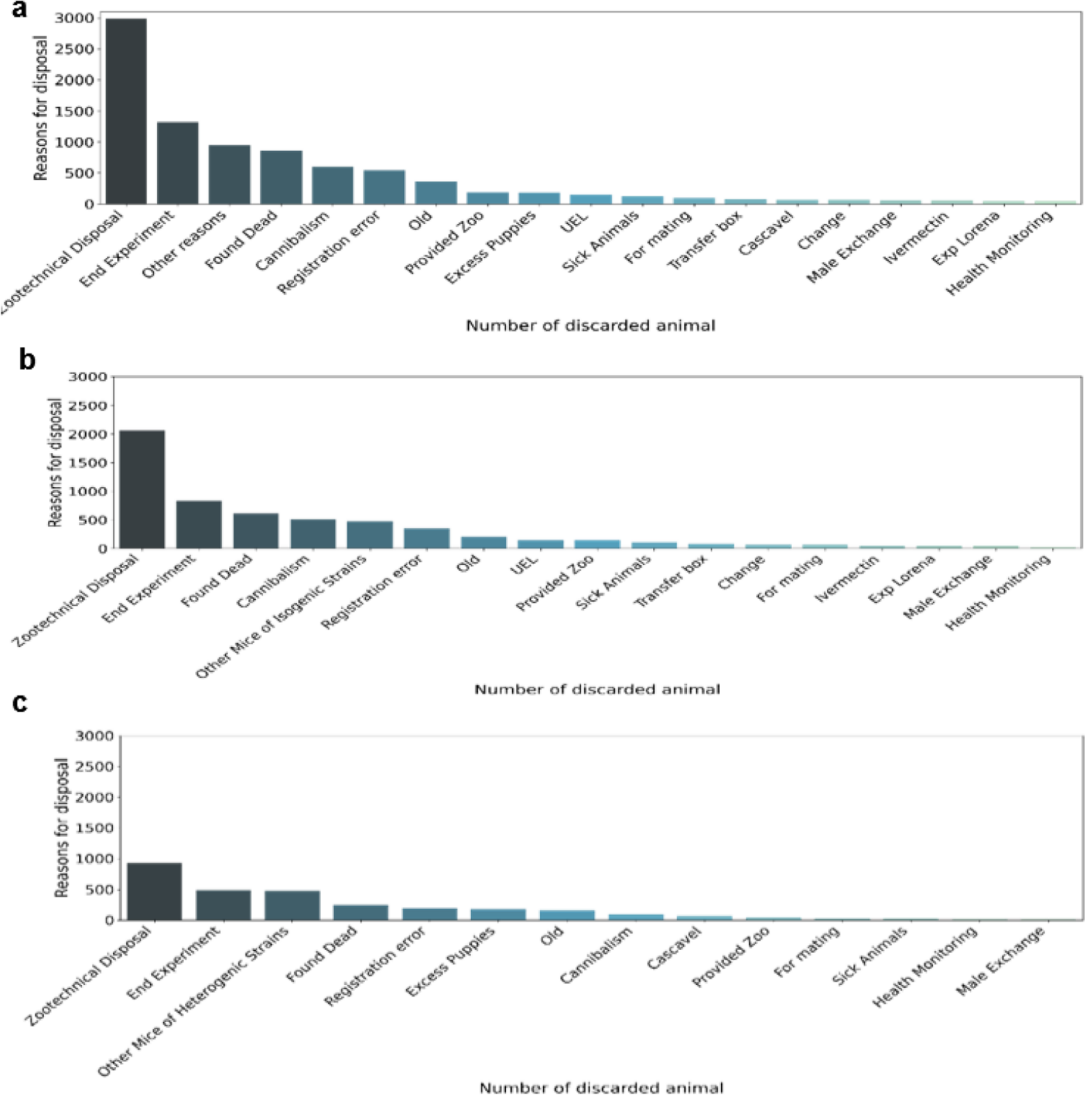
Total sum of animals that were discarded during the five-year period by LACEA. **a** Total animals that were discarded due to disposal among all the strains. **b** Graph showing the reasons for disposal in the isogenic group. The reason of zootechnical disposal is almost three times more common than disposal at the end of the experiment. **c** The graph presenting the reasons disposal indicates that disposals of the heterogenic group is lower than of the isogenic group

Figure 6a shows the sum of the discarded animals organized by reason of disposal, as recorded by BioterC users. Among all the reasons, the reason given for the highest number of animals is zootechnical disposal, which is a common reason for disposal at the laboratory animal facility. This reason is generally used for animals that have been discarded because they have not been used in experiments or if they have been overproduced.

In the isogenic group (Fig. 6b), 2061 animals were discarded by zootechnical disposal, with less than half maintained until the end of the experiment, suggesting that the other half of the mice produced were not used for the experiment. This can also be observed in the disposal of heterogenic animals (Fig. 6c); i.e., it was necessary to discard twice as many animals by zootechnical disposal than at the end of experimentation.

**Fig. 7:**
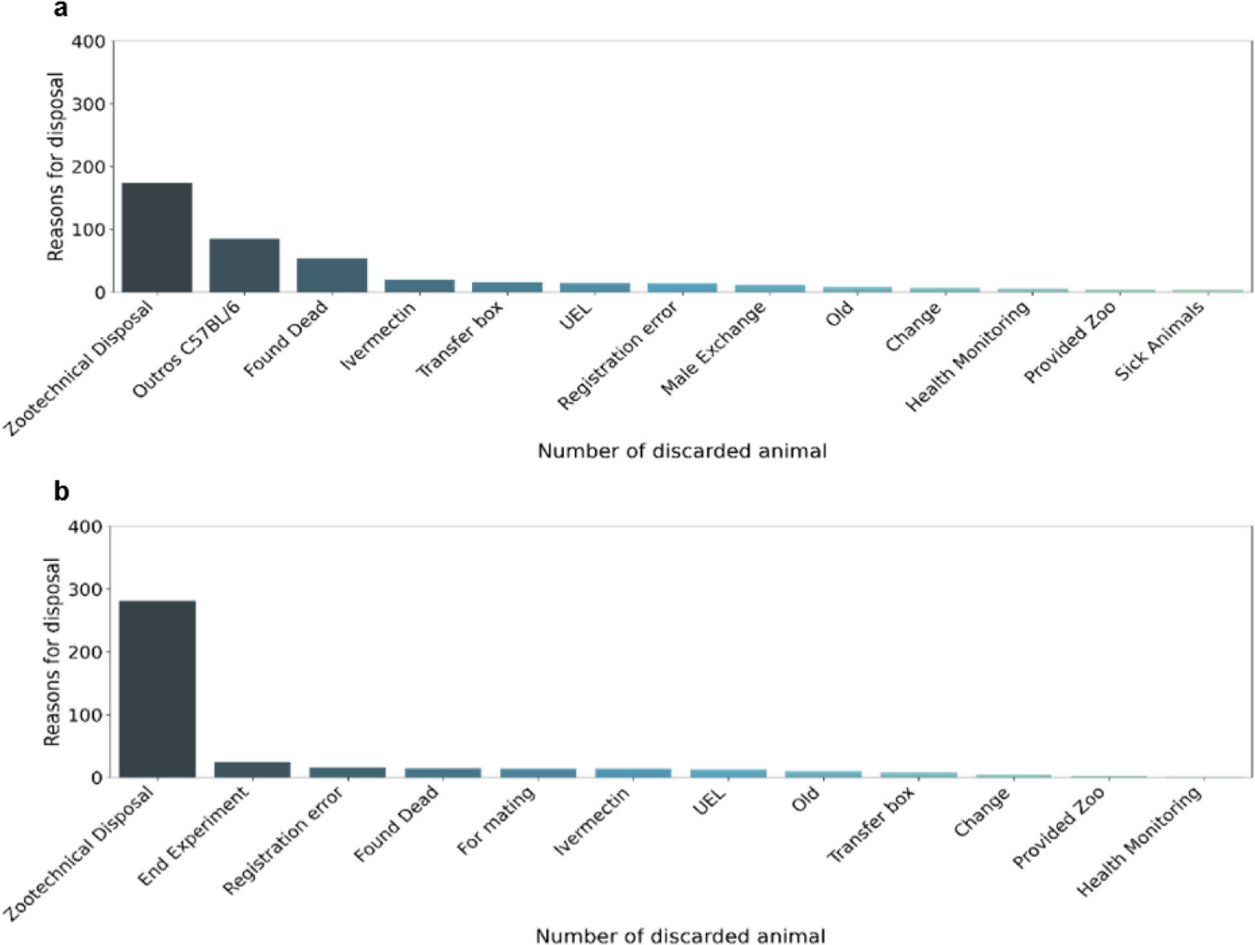
Reasons for C57BL/6 strain disposal. **a** Sum of the reasons for disposal of C57BL/6 male mice, with zootechnical disposal as the main reason. **b** Sum of the reasons for disposal of female C57BL/6 mice, with zootechnical disposal and the end of experimentation being the main reasons

After the analysis of disposal in the isogenic and heterogenic groups of animals, the disposal of C57BL/6 males and females was analyzed, since this is the most frequently produced strain in LACEA. In both conditions, when observing the 10 main disposal ratios in males (Fig. 7a) and females (Fig. 7b), the main reason was zootechnical disposal. In males, among the 10 main reasons for registration, there was no information on the end of experimentation, suggesting that male animals are not significantly used in experimentation. Conversely, in females, discarding at the end of experimentation is the second most common reason for disposal, corroborating the hypothesis. Additionally, females (281 animals) were subjected to zootechnical disposals 1.6 times more frequently than males (174 animals). The total production numbers of animals of this lineage that were recorded were 2293 male mice and 1706 female mice.

## Discussion

Among the analyses in the data set, we obtained relevant results for animal husbandry within a lab animal facility. Our findings confirmed breeding patterns, such as median births in the isogenic and heterogenic genetic constitution strains. In addition to identifying this breeding pattern, it was also possible to obtain results about animal disposal, which present discrepant values compared to production and request.

The results obtained through the AED, using the Data Science tool, facilitated the screening and analysis of the data, yielding results in a fast, easy and reliable way; in addition to being a free resource, the only necessary prior knowledge is familiarity with programming language. This tool can be applied in any study that requires analysis of a set of data and can also create time series and perform statistical analysis and production forecasts, among other approaches.

The first question was to determine, from the data, the number of animals that have already been produced in LACEA. From the records, the total number of mice produced was 15,106 mice among 11 different strains, with an annual average of 2220 animals. It was not possible to make a comparison from these data with another laboratory animal facility that presents production of mice similar to what was evaluated, since these data are not available for comparison of animal production, limiting our investigations.

As mentioned above, we obtained the median values for births in the different isogenic (brother x brother mating) and heterogenic (random mating) groups. Our results are consistent with Benavides [7] (2003), who states that isogenic strains usually give birth to 4 to 8 pups; in the case of some groups of (heterogeneous) mice, the number of pups can vary from 10 to 14, with 32 pups registered for a female group of Swiss mice (Swiss) [7]. According to Santos [5] (2002), the average numbers of offspring/calving are 8–10 in heterogeneous strains (i.e., outbred lines) and approximately 5 pups/calving in isogenic lines (i.e., inbred lines). Although the isogenic strains have fewer offspring, they are models that do not have genetic variability in the experiment and may reduce the number of animals that are used, and only the best model should be examined. Another question that we seek to answer through the data is the period between mating and the birth of mice reared in the laboratory animal facility. The data showed a period of 15 days longer than that observed by animal laboratory technicians. Santos [5] (2002) described the gestation period as ranging from 19 to 21 days; after the tenth day, the abdomens of females are already increasing in size [5]. Guénet [8] (2015) also states that the period between mating at birth ranged from 19 to 22 days in mice, varying by strain or stock. The duration of pregnancy, like most other aspects of mouse reproduction, depends on the lineage. For example, in isogenic DBA and C57BL strains, the duration of gestation is usually approximately 19–20 days [9]. To explain this discrepancy, the hypothesis was raised that due to the lower median of mouse births observed in the isogenic strains, it would take a longer time to generate greater offspring. When calculating the median mating period of isogenic mice alone, we obtained a median of 48.80 days, with heterogenic mice requiring 12 more days (36.51 days). Additionally, when analyzing the routine of the technicians in the lab animal facility, it is also believed that the records are being made outside the correct date.

After identifying the period between mating at birth, we determined the median period from birth to weaning. According to Santos [5] (2002), the sexing period starts from the weaning of the pups, which usually occurs after 19 days, and in isogenic lines, weaning can take place after 28 days [5]. At weaning, mice are separated by sex. Guénet [8] (2015) explains that breastfeeding lasts between 19 and 21 days, after which mice are ready for solid food and are ready to be sexed [8]. Even within the described period, the mean and median indicate the precocity of sexing. In terms of the behavior of pups and mothers separated prematurely, it was found that mice weaned early showed higher activity and lower behavior at rest from postnatal days 15 to 21, exhibiting signs of anxiety [10].

In addition, the requests that animal laboratory technicians receive for animal breeding and experimentation through BioterC were analyzed. The number of animals requested by external users is three times higher than that requested by internal users, but the requests occur less frequently. The most requested lineages among Carlos Chagas Institute users are BALB/c, Swiss and S129KO, with the BALB/c lineage being requested by most laboratories. The Swiss lineage ceased to be requested between 2018; from these data, the hypothesis was raised that this lineage was replaced by BALB/c, as researchers sought to reduce the use of animals and to refine their experiments.

An internal research was conducted in the laboratory animal facility, with the goal of profiling the external clients who request animals to understand the main reason for searching for LACEA mice. Among the data analyzed, 90% of the requests are from external research institutions [11]. The main reason for requests is due to the high availability of animals in the laboratory animal facility, in addition to the availability of quality animals.

Disposal is the last of the stages of animal husbandry and breeding. According to the guidelines for euthanasia established by CONCEA (National Council for Animal Breeding and Experimentation) (2013), the criteria often adopted for euthanasia recommendation in an individualized way are include injured animals, with impossibility of treatment, animals with terminal diseases experiencing intense suffering and elderly animals in the absence of resources to meet their needs. However, other situations that indicate the induction of death may occur, such as when animals are subjected to teaching or scientific research activities. Euthanasia requires moral and ethical considerations for the practice to be carried out in a humane manner [12].

The most frequent reason for disposal in the laboratory animal facility was zootechnical disposal, i.e., mice being discarded due to fighting, animals that are old and do not reproduce anymore, and mainly animals that were not used for experiments or animals created in excess. Cardoso [13] (2002) shows that considering the condition of the vivarium, which aims for high productivity at the lowest possible cost, the disposal of undesirable animals being a necessary measure [13].

Based on the data, zootechnical disposal is the most common reason for animals being discarded, as represented by 2986 euthanized animals in this dataset (7.889 total sum). In addition, twice as many animals were discarded compared to those sacrificed the end of experiments. When searching the isogenic and heterogenic animals, both groups included instances of zootechnical disposal that were substantially higher than the instances of animals being used for experimentation, suggesting that it is necessary to produce a certain fold excess of animals to meet the demands for animal experimentation.

After analyzing the discarded numbers in the groups of animals, we also determined the rate of discarded for the lineage most produced by LACEA. As verified in the order data, the C57BL/6 strain is the most frequently produced and requested lineage by external users, while few animals were requested among internal users. The C57BL/6 strain had a total production of 3553 mice, and 1780 were ordered, including males and females – half of which were produced to provide the requested number. This represents another group of data that could not be compared with laboratory animal facilities from other institutions due to the difficulty of accessing this information.

By tracing the parameters of animal production and disposal, our results allow a discussion about the “The Principles of Humane Experimental Technique” (i.e., the 3 Rs - Replace, Reduce and Refine), which include replacing the use of animals with alternative models whenever possible. These principles also include the reduction of the number of animals per experiment, without harming the quality of the experimental results, which suggests the use of isogenic animals; even if lower offspring numbers are produced, the genetic variability is lower, reducing the number of animals required, and maintaining awareness of the number of animals that are discarded to meet the demand for requests [14].

Among the difficulties encountered is the lack of current references for comparisons, as this is a recently developed technique for analysis that is rarely found among scientific research and is instead often used in exact areas.

## Conclusions

Based on our findings, it was possible to compare mouse breeding patterns with what is already observed in practice, showing positive results. In addition, it was possible to observe the excess of animal waste that is not used during the experiment. Opening the discussion on the principles of 3 R’s, contributing to Replace, Reduce and Refine.

Future directions of this work are to reanalyze this set of data to generate time series and determine how these data behave within each period of time analyzed. In addition, future work will generate predictive models of mouse production to be able to predict production for the coming years and to determine the gains or losses for the institution in order to facilitate decisions about maintaining or removing strains.

## Methods

### Python libraries

The methodological approach used was Data Science, aiming to analyze the set of data obtained from the BioterC software from 2014 to 2019. The Python programming language was used to develop exploratory data analysis (EDA) tools. The Python language was chosen because a number of computational features, such as modules and libraries prepared for EDA are available. Different libraries were used as tools for analysis and high-performance data structures, such as Pandas, Numpy, Matplotlib and Seaborn.

### Data science

**Fig. 8:**
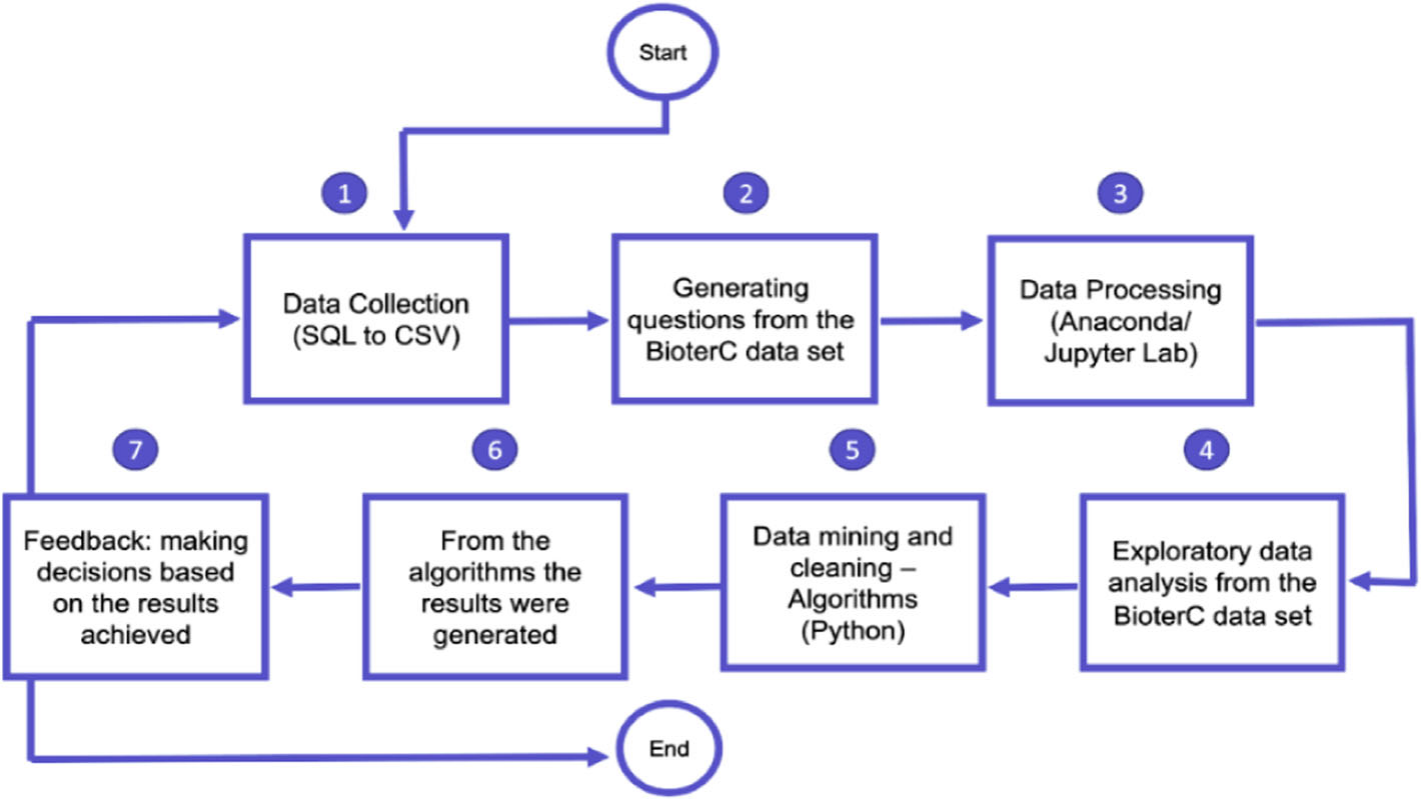
Flowchart of BioterC data processing. Steps taken during the exploratory analysis of the data set

The BioterC data were initially organized in a Structured Query Language (SQL) and were converted to a CSV file (Comma-Separated Values) for analysis. The analysis environment was prepared with the aid of the freely distributed Anaconda tool, which allows access to the Jupyter, Lab Integral Development Environment (IDE) (Fig. 8).

### Logic for generating algorithms

To work with datasets that were arranged in tables, it was necessary to access them through a network database in SQL format and convert them to CSV. To structure the information, the Pandas library was imported, and a DataFrame (rows and columns) was created from one of the tables contained in the data.

#### Total sum of mice per strain

To calculate the sum of all the mice that were registered in BioterC in the period in which it was used (Sep 2014 to Jun 2019), only the column with the number of animals was filtered, and these values were added through the sum() function. To visualize these data, they was plotted on a bar chart, for which it was necessary to import the Seaborn library and use the barplot() method from Matplotlib*.* To visualize the sum of mice among all registered strains, the groupby() method was used*,* and this method has the function of grouping the data according to the parameter used; in this case, we passed the column of the strains. To calculate the average of animals, the mean() function was used in the grouped data.

#### Median births in the isogenic and heterogenic groups

To determine the median births in the groups of different genetic background, first, two lists were created: one list included strains that are isogenic, and the other list included strains that are heterogeneous. Then, we searched the DataFrame for the strains contained in the lists using the isin() method. Separate graphs were generated to analyze the median of the values using median() for each lineage within each group, again using the plot() and groupby() methods to group by lineages. To plot the mean and median intervals, these values were calculated again based on the median births.

#### Median between periods

To analyze the periods between mating at birth and after birth to sex differentiation of mice (sexing), it was necessary to filter the columns where the start dates are recorded at the end of each period. From these values, the start date was subtracted from the end date, yielding the days in this period; a new column containing these values was created, and the median was calculated. The mean and median ranges were calculated in the same way as mentioned above.

#### Lineages most frequently requested by internal and external users

To analyze the request data, it was necessary to use another table in the dataset, which contained supply records. This table contains information about internal and external users. To facilitate our analysis, these data were first filtered only for information from internal users, and a DataFrame was created from these data. The data were grouped by strains, and the values were summed using sum() to facilitate the identification of the most requested strains. To search for the strains most frequently supplied to external users, the same logic was used.

#### Reasons for disposal of mice

The discard dataset has a part field, in addition to having fixed disposal options; it also has a feature that allows the user to enter the reason why the mouse was being discarded. Because this field is an open field for typing, it was necessary to process and mine the data. After this screening, the data still indicated different reasons for disposal; 90% of the data were separated, and another 10% were concatenated and renamed “others” for easy visualization. To perform the calculation, the groupby function was again used*,* but in this case, the data were grouped by the discard reasons column, and the values were summed using sum(). For the C57BL/6 lineage, it was necessary to create a DataFrame*,* filtering information only from this lineage and grouping it again. To better visualize the data, we plotted them on a bar chart.

## Data Availability

The dataset(s) supporting the conclusions of this article is(are) available in the Dataset_BMC repository in GitHub, [https://github.com/eloiza-dias/Dataset_BMC].

## Abbreviations

CSV: Comma-Separated Values
CONCEA: National Council for Animal Breeding and Experimentation
EDA: Exploratory Analysis Data
IDE: Integral Development Environment
INPI: National Institute of Industrial Property
LACEA: Laboratório de Criação e Experimentação Animal
LAS: Laboratory Animals Science
SPF: Specific Pathogen-Free
SQL: Structured Query Language
